# Postcaesarean Pseudoaneurysm of Uterine Artery Causing Secondary Postpartum Hemorrhage Managed With Embolization: A Case Report

**DOI:** 10.1155/crog/4977602

**Published:** 2026-07-24

**Authors:** Shova Aryal, Sangit Adhikari, Kashish Kumari Sah, Asmita Ghimire, Poonam Koirala, Hima Rijal

**Affiliations:** ^1^ Department of Obstetrics and Gynecology, Maharajgunj Medical Campus, Institute of Medicine, Tribhuvan University, Kathmandu, Nepal, tribhuvan-university.edu.np; ^2^ Department of Obstetrics and Gynecology, Tribhuvan University Teaching Hospital, Kathmandu, Nepal, teachinghospital.org.np

**Keywords:** case report, cesarean section, embolization, postpartum hemorrhage, pseudoaneurysm

## Abstract

**Background and Aims:**

Secondary postpartum hemorrhage (SPPH) is defined as the hemorrhage occurring beyond 24 h and within the puerperium (6 weeks). The incidence of SPPH is 0.21%. Pseudoaneurysm of the uterine artery is a rare cause of SPPH following the caesarean or vaginal delivery and is reported to be developed as a complication of vascular trauma during caesarian section or after uterine curettage and pelvic surgery. Prompt diagnosis and early management should be done to avoid the complications.

**Methods:**

An informed written consent was taken from a patient for writing the case. A brief literature review was done for writing the case.

**Results:**

In our case, the patient had recurrent vaginal bleeding following LSCS where she was managed conservatively along with multiple blood transfusions. The final diagnosis was made on the 48th day postpartum. This highlights the necessity of considering further investigation to rule out the rare causes of SPPH like pseudoaneurysm and also emphasizes the role of TAE in the management of such cases.

**Conclusion:**

Early diagnosis and management of SPPH is crucial to reduce complications in patients. Pseudoaneurysm should be ruled out, though rare in patients presenting with recurrent vaginal bleeding in the postpartum period. Pseudoaneurysm can be effectively managed with the TAE considering its advantages.

**Highlights**

• Pseudoneurysm is a rare cause of secondary postpartum hemorrhage.
• CT angiography is the gold standard for the diagnosis of pseudoaneurysm.
• Transarterial embolization is the less invasive and effective method for the management of the pseudoaneurysm.
• Hysterectomy should be a last resort for PPH.

## 1. Introduction

Clinically, postpartum hemorrhage (PPH) is defined as any amount of bleeding from or into the genital tract following birth of the baby up to the end of puerperium, which adversely affects the general condition of the patient evidenced by rise in pulse rate and falling blood pressure. World Health Organization (WHO) defines PPH as the amount of blood loss in excess of 500 mL following the birth of the baby. Primary PPH is the hemorrhage occurring within 24 h of delivery. Secondary postpartum hemorrhage (SPPH) is defined as the hemorrhage occurring beyond 24 h and within the puerperium (6 weeks) [1]. SPPH is also called as delayed or late puerperal hemorrhage. The incidence of SPPH is 0.21% [2]. It is the most common indication for the readmission after delivery [2]. It causes significant mortality and morbidity and can cause serious complications when prompt diagnosis and treatment are not made [2]. Commonly, it occurs due to retained product of conception (RPOC), infection, endometritis, or subinvolution of uterus, and rarely due to a pseudoaneurysm of the uterine artery, arteriovenous malformations, or choriocarcinoma [3]. Pseudoaneurysm of the uterine artery is the rare cause of SPPH following the caesarean or vaginal delivery and is reported to be developed as a complication of vascular trauma during caesarian section or after uterine curettage and pelvic surgery [4]. Diagnosis is usually made by doppler sonography and confirmed by uterine artery angiography [5]. Management is based on the amount of blood loss and cause of SPPH. Secondary hemorrhage following caesarean section may at times require laparotomy or other interventions.

Herein, we report a case of uterine artery pseudoaneurysm presenting with SPPH in a 31‐year‐old female on the 48th day after lower segment caesarean section (LSCS) delivery which was managed with uterine artery embolization.

## 2. Case Presentation

### 2.1. Case History and Examination

A 31 year old Para 2 Living 3 (P2L3) female presented to the emergency room with complaints of excessive vaginal bleeding for three days on her 48th postpartum day (which was the third episode). She had undergone an emergency LSCS for oligohydramnios and was discharged uneventfully 4 days postoperatively. She had similar episodes of vaginal bleeding on the 14th (first episode) and 27th (third episode) postoperative days, which was managed conservatively along with blood transfusions. This was her third episode, leading to her referral to our center for further management. On first episode, there was around 1000 mL of blood loss, which was managed with transfusion of packed red blood cells and intravenous fluids and other medications. Investigations and imaging were done. In the second episode, she had around 800 mL of blood loss, which was managed similarly as first episode.

On arrival, she complained of vaginal bleeding and generalized body weakness. She complained of bleeding around 600 mL of blood loss. She had no fever, wound discharge, bleeding from other sites, history of trauma, or breast discomfort. She was experiencing lactational amenorrhea but previously had normal menstrual cycles. Her obstetric history includes a previous caesarean delivery of male twins 5 years ago and a recent LSCS delivery of a female child 48 days ago. Her past medical history includes a cholecystectomy 5 years ago and hypothyroidism, for which she has been on medication for 5 years.

On general examination, she was ill‐looking, moderately built, having pallor, and afebrile; however, her vitals were stable. A healthy suprapubic scar was seen, the abdomen was soft, nontender, nondistended, and the uterus was not palpable. Chest and cardiovascular system examination were normal. Per speculum examination, the cervix and vagina were normal‐looking, with no discharge and no active bleeding. Per vaginal examination, the uterus was anteverted, nontender, mobile, the os was closed, bilateral adnexa were free, and cervical motion tenderness was absent.

### 2.2. Investigations and Diagnosis

The laboratory results in were as follows: Hb: 11.5gm%, PCV: 32.1gm%, platelets: 1.25million/cu, TLC: 4200/cmm, DC: N55L31E8M6, PT: 15 sec, INR: 1.25, CRP (quantitative): 14.56 mg/L, RBS: 5 mmol/L, serum creatinine: 42 *μ*mol/L, serum Na+: 138 mEq/L, and serum K+: 1.3 mEq/L. Ultrasonography (USG) of the abdomen and pelvis showed minimal fluid collection in the uterine cavity without internal vascularity as shown in figure 1. USG Doppler showed the “yin‐yang” swirling flow with a to‐and‐fro spectral waveform at the communicating neck, confirming a uterine artery pseudoaneurysm. Computed tomography (CT) angiography showed the focal outpouching filled with contrast arising from the left iliac artery in the left uterine artery as shown in figure 2, 3, and 4. This confirmed the presence of a uterine artery pseudoaneurysm with surrounding hematoma, communicating with the uterine cavity.

**Figure 1:**
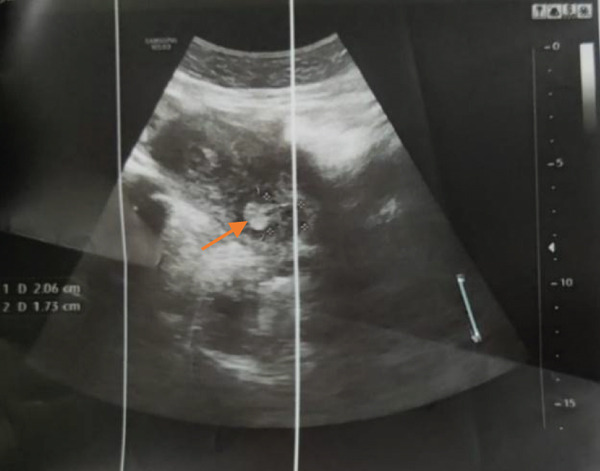
Ultrasonography of the abdomen and pelvis showed minimal fluid collection in the uterine cavity without internal vascularity, indicated by an arrow.

**Figure 2:**
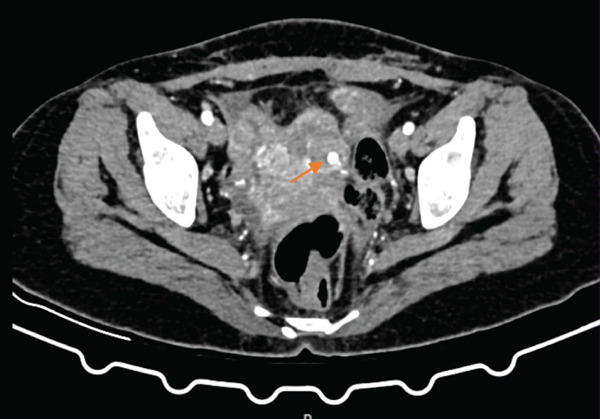
CT angiogram axial view showing focal outpouching filled with contrast surrounding a hematoma in the left side, indicated by an arrow.

**Figure 3:**
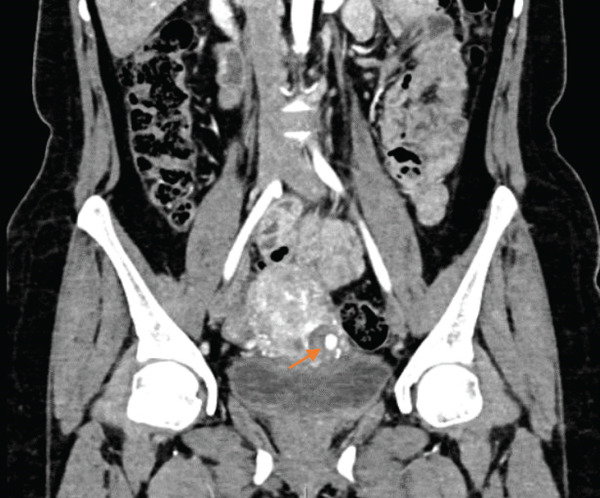
CT angiogram coronal view showing contrast filling the pseudoaneurysm, indicated by an arrow.

**Figure 4:**
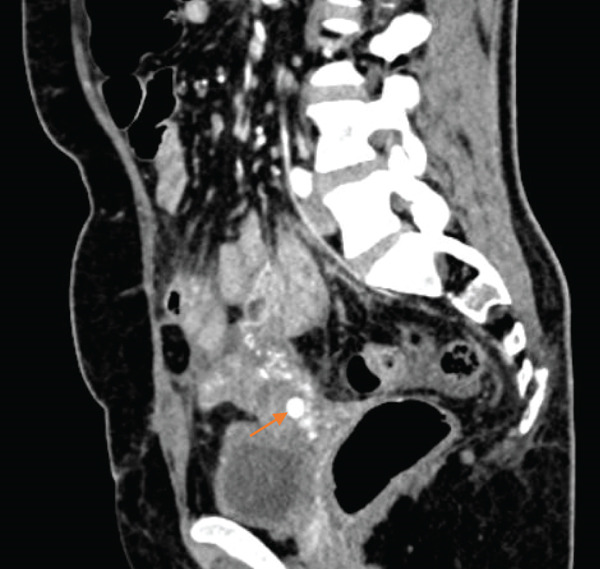
CT angiogram sagittal view showing pseudoaneurysm filled with contrast, indicated by an arrow.

### 2.3. Management

Conservative management included intravenous fluids, antibiotics, and blood transfusion. Antifibrinolytic agents were given. Planned left uterine artery pseudoaneurysm embolization was performed successfully under fluoroscopic guidance with digital subtraction angiography (DSA) shown in figure 5(a) and 5(b); the left uterine artery was accessed via left iliac artery through the right femoral artery. Liquid embolicus (0.2 mL glue and 0.4 mL lipiodol) was used for the embolization, and obliteration of the pseudoaneurysm was achieved shown in figure 6. Hematoma was secured with compression and a pressure bandage. The patient had an uneventful hospital stay and showed positive progress during normal follow‐up.

**Figure 5:**
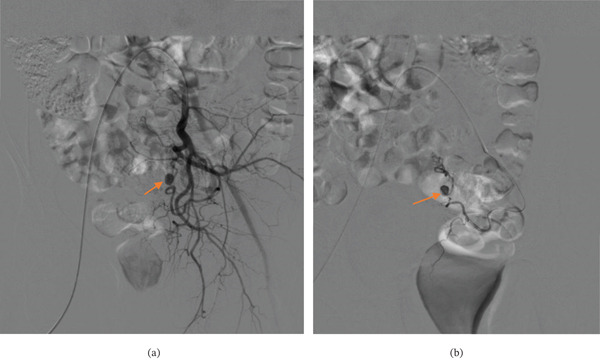
(a,b) Digital subtraction angiography (DSA) of the left internal iliac artery showing pseudoaneurysm of the left uterine artery (arrow).

**Figure 6:**
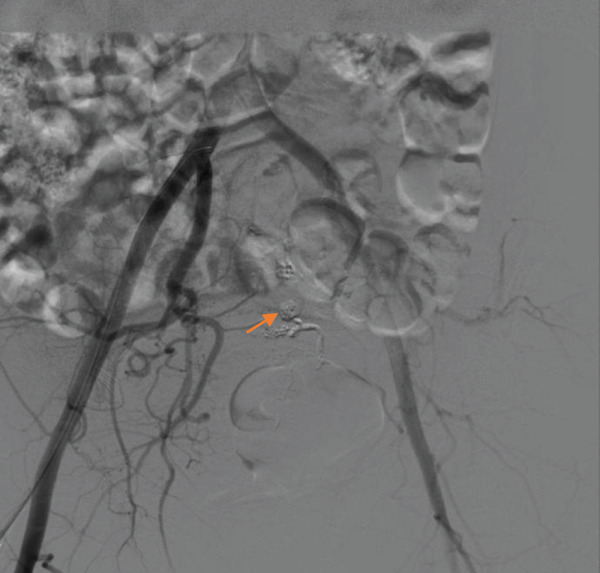
Digital subtraction angiography (DSA) of the left internal iliac artery (postembolization) using liquid embolics and embolization coils show complete obliteration of the pseudoaneurysm (arrow).

## 3. Discussion

SPPH is defined as the hemorrhage occurring beyond 24 h and within 6 weeks postpartum [1]. It most commonly occurs between 8 and 10 days postpartum [4]. It causes significant mortality and morbidity and can cause serious complications when prompt diagnosis and treatment are not made [2, 4]. Pseudoaneurysm (also known as false aneurysm) is created when blood is allowed to enter the periarterial tissue in a lacerated arterial wall which then creates a profuse sac communicating with the parent arterial lumen [5]. Pseudoaneurysm formation is uncommon in young women and is commonly associated with trauma, pelvic surgery, neoplasm, infection, or caesarian section [6]. Caesarean section is the most common cause of uterine artery pseudoanerysm [7]. Pseudoaneurysm has also been reported following hysterectomy, myomectomy, and even uncomplicated vaginal delivery. http://samad.et.al/ has reported a massive PPH 7 weeks after a caesarean section caused by a pseudoaneurysm of the uterine artery [6]. It can present as late as 2 years and delayed diagnosis may be life threatening [7].

In present case we report the SPPH due to pseudoaneurysm which was confirmed with the CT angiography. Chaware et.al [5] had reported a SPPH as a result of pseudoaneurysm of uterine artery at D36 following the caesarian section. Chitra and Panicker [8] presented a case series of five women who underwent LSCS and had SPPH due to pseudoaneurysm of uterine artery and were treated by embolization.

Although being a rare cause of PPH, pseudoaneurysm should be thought of as it may cause mortality if timely diagnosis is not made. In pseudoaneurysm, bleeding may occur repeatedly as they cannot endure the turbulent blood flow extravascularly, which may cause massive extravasation any time [9]. The pseudoaneurysm can be prevented during caesarian section with measures like proper securing of hemostasis with sutures and stepwise devascularization to avoid the arterial injury.

The diagnosis of pseudoaneurysms can be made with USG, color Doppler (characteristic swirling flow seen), CT scan, and CT angiography. USG with color Doppler is an easily available means of diagnosis and has a higher sensitivity. In our case, the USG Doppler showed the “yin‐yang” swirling flow with a to‐and‐fro spectral waveform at the communicating neck, confirming a uterine artery pseudoaneurysm. This “yin‐yang” in the USG Doppler strongly suggests pseudoaneurysm [4]. It is confirmed by CT angiography as it remains the gold standard for the diagnosis of the pseudoaneurysm. CT or magnetic resonance imaging (MRI) often helps in differentiating findings of pseudoaneurysm on color Doppler from other abnormalities like arteriovenous malformations or fistulas [9].

In patients presenting with the complaints of bleeding, CT angiography should be considered as it helps in identifying less common areas of hemorrhage outside of the uterus and vagina by providing a wide view of pelvis [10]. Treatment modalities for pseudoaneurysm include hospitalization and observation, endovascular embolization, and hysterectomy [11].

Embolization as a method of treatment is new and is evolving as an alternative method to open methods. The transcatheter arterial embolization method as treatment was first reported and described in 1979 by Wu et.al [12] for the multiple failed surgical attempts for the management of pelvic hematoma. Nowadays, almost all the cases reported have been managed with the embolization. Uterine artery embolization is an effective and safer method of treatment of postpartum hemorrhage as it preserves the patient′s reproductive function [13]. There are many advantages of embolization, and the success rate is found to be about 97%. Some advantages are its fast pace, good success rate, minimal invasiveness, and avoids the need for general anesthesia. The exact bleeding site can be identified and treated, the fertility of the patient is preserved, and it does not cause hemorrhage unlike open procedures, and if needed, re‐embolization can be performed easily [8]. Sometimes bilateral uterine artery embolization may be needed due to redistribution from the opposite side uterine artery. However, re‐embolization or open surgical management may be needed sometimes if collateral circulation is present in the pseudoaneurysm. Also, as this procedure requires an interventional radiologist (IR), its application is limited to only centers with IR. Many studies recommend considering transarterial embolization (TAE) as the first‐line therapy to control PPH due to its multiple advantages and good success rate [14].

## 4. Conclusion

Early diagnosis and management of SPPH is crucial to reduce complications in patients. It is important that obstetricians and gynecologists should think of the pseudoaneurysm, although being rare, when women come with the complaint per vaginal bleeding postpartum, and should do further investigation when the common causes have been ruled out. Hysterectomy should be a last resort for PPH. TAE is a preferred first‐line treatment for its benefits and success rate.

**Nomenclature**
(LSCS): lower segment caesarean section.
(PPH): postpartum hemorrhage.
(RPOC): retained product of conception.
(SPPH): secondary postpartum hemorrhage.
(USG): ultrasonography.
(WHO): World Health Organization.

## Funding

No funding was received for this manuscript.

## Disclosure

Asmita Ghimire affirms that this manuscript is an honest, accurate, and transparent account of the study being reported; that no important aspects of the study have been omitted; and that any discrepancies from the study as planned (and, if relevant, registered) have been explained. All authors have read and approved the final version of the manuscript. The corresponding author had full access to all of the data in this study and takes complete responsibility for the integrity of the data and the accuracy of the data analysis.

## Consent

Written informed consent was obtained from the patient for publication of this case report and accompanying images. A copy of the written consent is available for review by the editor‐in‐chief of this journal upon request.

## Conflicts of Interest

The authors declare no conflicts of interest.

## Data Availability

All the data generated or analyzed during this study are included in the manuscript.
